# Robotic tests for position sense and movement discrimination in the upper limb reveal that they each are highly reproducible but not correlated in healthy individuals

**DOI:** 10.1186/s12984-020-00721-2

**Published:** 2020-07-25

**Authors:** Catherine R. Lowrey, Benett Blazevski, Jean-Luc Marnet, Helen Bretzke, Sean P. Dukelow, Stephen H. Scott

**Affiliations:** 1 Laboratory of Integrative Motor Behaviour, Centre for Neuroscience Studies, Queen’s University, 18 Stuart St., Kingston, ON K7L 3N6 Canada; 2 BioEngineering and Innovation in Neuroscience, University Paris Descartes, Paris, France; 3 Hotchkiss Brain Institute, University of Calgary, Calgary, Alberta Canada; 4 Department of Biomedical and Molecular Sciences, Queen’s University, Kingston, ON Canada; 5 Department of Medicine, Queen’s University, Kingston, ON Canada

## Abstract

**Background:**

Robotic technologies for neurological assessment provide sensitive, objective measures of behavioural impairments associated with injuries or disease such as stroke. Previous robotic tasks to assess proprioception typically involve single limbs or in some cases both limbs. The challenge with these approaches is that they often rely on intact motor function and/or working memory to remember/reproduce limb position, both of which can be impaired following stroke. Here, we examine the feasibility of a single-arm Movement Discrimination Threshold (MDT) task to assess proprioception by quantifying thresholds for sensing passive limb movement without vision. We use a staircase method to adjust movement magnitude based on subject performance throughout the task in order to reduce assessment time. We compare MDT task performance to our previously-designed Arm Position Matching (APM) task. Critically, we determine test-retest reliability of each task in the same population of healthy controls.

**Method:**

Healthy participants (*N* = 21, age = 18–22 years) completed both tasks in the End-Point Kinarm robot. In the MDT task the robot moved the dominant arm left or right and participants indicated the direction moved. Movement displacement was systematically adjusted (decreased after correct answers, increased after incorrect) until the Discrimination Threshold was found. In the APM task, the robot moved the dominant arm and participants “mirror-matched” with the non-dominant arm.

**Results:**

Discrimination Threshold for direction of arm displacement in the MDT task ranged from 0.1–1.3 cm. Displacement Variability ranged from 0.11–0.71 cm. Test-retest reliability of Discrimination Threshold based on ICC confidence intervals was moderate to excellent (range, ICC = 0.78 [0.52–0.90]). Interestingly, ICC values for Discrimination Threshold increased to 0.90 [0.77–0.96] (good to excellent) when the number of trials was reduced to the first 50. Most APM parameters had ICC’s above 0.80, (range, ICC = [0.86–0.88]) with the exception of variability (ICC = 0.30). Importantly, no parameters were significantly correlated across tasks as Spearman rank correlations across parameter-pairings ranged from − 0.27 to 0.30.

**Conclusions:**

The MDT task is a feasible and reliable task, assessing movement discrimination threshold in ~ 17 min. Lack of correlation between the MDT and a position-matching task (APM) indicates that these tasks assess unique aspects of proprioception that are not strongly related in young, healthy individuals.

## Background

Proprioception can be divided into three distinct percepts: position sense, kinesthesia (sense of motion) and sense of effort [1, 2]. The two former percepts are predominantly provided by primary and secondary muscle afferents, although cutaneous afferents also play a role, particularly related to the hand [3, 4]. These sources of sensory information travel to the primary somatosensory cortex via the dorsal column-medial lemniscus pathway and through the ventral posterior lateral nucleus of the thalamus [5]. Beyond primary somatosensory cortex, there are several other cortical regions that have been implicated in proprioceptive function [6–9].

When quantifying impairments following stroke, the assessment of motor function has been the dominant focus due to the common impact that stroke has on one’s ability to move and interact in the world. Far less attention has been placed on tools to quantify impairments in sensory function, even though sensory function is commonly impacted following stroke [10, 11] and linked to poor functional recovery [12]. Some clinical tools have been developed to assess somatosensation, such as the Nottingham Sensory Assessment protocol [13]. However, proprioceptive function is commonly assessed simply by having an individual close their eyes while passively moving their finger up or down, and asking them if they can identify the direction of movement [14]. For the proximal arm, a Thumb Localizer Task has been developed where the individual shuts their eyes and an examiner moves their hand to a randomly chosen location and then the individual must attempt to grasp their thumb with their opposite hand [15]. Unfortunately, these types of scales lack sensitivity, can show poor inter-rater reliability and lack specificity [16, 17] (however, see [13]).

Robotic technologies are emerging as a new approach for neurological assessment. They offer advantages over existing clinical tools as they can provide objective and precise measures of many sensory, motor and cognitive behaviours [18–20]. In recent years, there have been several new tasks developed using robotic technology for evaluating different aspects of proprioception including position sense [10, 20–27], sense of effort [28] and kinesthesia [29]. Many of these studies highlight that impairments in proprioception are common following stroke with highly variable patterns of recovery [11]. Importantly, such impairments slow functional recovery [12].

There are two common approaches for quantifying proprioception. The first approach involves passively moving the limb to a specified location and then the subject mirror matches this position with the other arm without vision [10]. The potential caveat with this approach is the assumption that proprioceptive and motor function of the other arm is not impaired. However, ipsilesional impairments can be observed in some individuals (~ 30%) following stroke [30–34]. Further, bilateral impairments are common in other diseases such as ALS [35–37]. The second approach is to passively move the subject’s arm to a specified target position and then back to the ‘start’ position. The subject is then asked to actively move their limb to the same location. This removes the influence of the other arm, but requires the use of working memory [38, 39] and sufficient motor function to move the arm to the specified location, both of which may be impaired following stroke [40, 41].

An alternate approach is to assess proprioception by quantifying the threshold for sensing limb movement, an aspect of kinesthesia. In this approach, proprioceptive acuity is typically measured as the threshold for detecting a difference between two movements [42, 43] or the detection of the onset of passive movement of the limb [44, 45]. Advantages to this approach are the fact that it does not rely on the use of the contralateral limb or as much on working memory, both of which could confound results. However, a major challenge with these tasks is that they commonly can take a long time to complete. Typically, a large set of different positions and/or speeds must be assessed for the algorithms used to calculate detection thresholds, commonly resulting in total assessment times of up to 45 min [22, 43, 46].

Here we develop a movement discrimination task to quantify the threshold for discriminating movement of the arm. In order to reduce the amount of time to complete the task, we implement a staircase method to adjust movement magnitude based on the subject’s performance. We present the performance of young, healthy controls to determine the feasibility of the technique to determine a proprioceptive threshold. We also examine the test-retest reliability of the task.

Finally, we compare performance on this movement discrimination threshold task to a standard arm position matching (APM) task in which the robot moves one arm and the subject must mirror match with the other arm. Our working hypothesis is that performance in these two tasks should be correlated. Intuitively, it seems plausible for movement discrimination to be highly related to accuracy in a position matching task: the better you are able to detect small differences in positions or movements, the more accurate you would be able to match the position or motion of your arm. Further, similar cortical regions are involved in position sense and kinesthesia [9]. However, previous work, primarily in the lower limb, has found that position sense in healthy populations is not correlated with measures such as the threshold for detecting passive movement [47, 48] or the “just noticeable difference” between two positions [42]. The problem with many of these previous studies is that they do not report the test-retest reliability of the proprioceptive testing methods for the same population of subjects. In order to make conclusions about the relationship between the performances on two proprioceptive tests, we perform a test-retest on both tasks so that we can determine whether variability in performance across tasks reflects differences due to natural performance variability or due to the differences between the two proprioceptive tasks.

## Methods

### Participant recruitment

Participants were recruited from the student population at Queen’s University. Participants were included in the study if they were 18 years of age or older, and could understand task instructions. They were excluded if they had any neurologic or musculoskeletal diagnoses affecting the upper limbs. This study was approved by Queen’s University Research Ethics Board, which adhered to the principles of the Canadian Tri-council Policy statement on Ethical Conduct for Research Involving Humans and the principles of the Declaration of Helsinki (1964). All participants provided consent to participate in the study and were free to stop participating in the study at any point.

### Robotic assessment

**Fig. 1:**
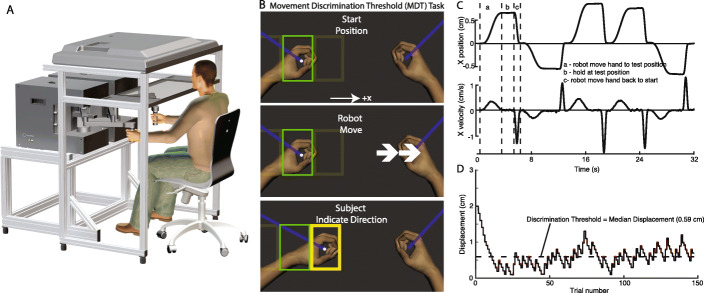
**a** Kinarm Endpoint Lab **b** Schematic of the Movement Discrimination Threshold (MDT) task. Subjects were seated and grasped the handles of the robot and the arms were occluded from view. Robot moved dominant hand and subjects used the opposite arm to indicate the direction of movement (right or left) by moving into the corresponding rectangular target (arms were occluded from view, subjects could see the rectangles on the screen and the white hand feedback dot indicating the position of the non-dominant hand). **c** Example traces of hand displacement (X position) and velocity (X Velocity) of the robot-moved dominant arm. **d** Example of displacements imposed on the arm during the MDT task. Commanded displacements are displayed in red and the actual displacements of the hand are displayed in black. Displacements began at 2 cm and were reduced by 1 step for each correct answer and increased by 3 steps after an incorrect answer. Discrimination Threshold was determined as the median of all displacements after the first wrong answer

Robotic assessments were performed using the Kinarm End-point lab (Fig. 1; Kinarm, Kingston, Ontario, Canada; http://www.kinarm.com/bkin-products/kinarm-end-point-lab/). Participants were seated in a wheeled chair and moved in towards the workspace, after which the wheels were locked in place. The participants grasped the robotic handles to perform each task. During all tasks the participant’s arms were occluded from their view. Experimental set-up and robotic testing were completed on the same day by the same examiner for all participants and tests were completed in the same order for each participant. We chose to examine performance on the same day to minimize factors such as mood and fatigue changes for across-task comparisons, and thus, also chose to perform within-day test-retest reliability. Participants completed two repetitions of the Arm Position Matching (APM) task followed by two repetitions of the Movement Discrimination Threshold (MDT) task. Each task tested proprioception of the dominant arm. Results from the MDT task are presented first.

### Experiment 1

In the MDT task the robot moved the participant’s dominant arm to the left or to the right and participants were instructed to make a two-alternative forced-choice decision as to which direction the arm was moved. Arm dominance was self-reported by the participants. Participants indicated the direction of movement with their non-dominant arm, which was visible as a white dot representing the center of the handle. To begin each trial, participants were instructed to place the white dot in the middle segment of a rectangle before the beginning of each trial (Fig. 1b). The first 3 trials were training trials where a red dot moved across the screen (with no robotic manipulation of the arm) and participants indicated which direction the dot moved by moving their non-dominant arm to the left or right. The task then consisted of 150 trials (153 trials total) where the passive arm was displaced either right or left. The participant then indicated the direction of dominant hand motion by moving their non-dominant arm to the left or right. To successfully indicate a direction of movement, participants had to move the non-dominant arm outside a green rectangle on the screen (rectangle size was 10 cm x-direction and 15 cm y-direction) to highlight one of the adjacent rectangles and log the direction of perceived movement (See Fig. 1b).

Exemplar movement of the arm is shown in Fig. 1c. The arm was moved over a 3 s period using a bell-shaped velocity profile and then held at the new location. Over all movements, for all participants, peak movement velocities ranged from 0.084 ± 0.08 cm/s to 1.6 ± 0.79 cm/s (peak of bell-shaped velocity profile) for the smallest to the largest displacements, respectively. The number of left and right displacements were determined by a uniformly distributed randomly generated value between 0 and 1. If the value was < 0.5 the robot moved the hand to the right; otherwise it moved the hand to the left. Across subjects, actual distributions of displacements ranged from 60 to 40% for a given arm.

The passive arm was initially moved 2.0 cm and the size of the displacement was decreased by 1 “step” after every correct response and increased by 3 steps after every incorrect response (Fig. 1d). The step size was determined by the previous displacement size. When displacements were between 1.0 and 2.0 cm, the step sizes were 0.2 cm, meaning after a correct response the displacement was decreased by 0.2 cm and after an incorrect response it was increased by 0.6 cm. When the displacement fell below 1.0 cm, the steps decreased to 0.1 cm (displacements decreased by 0.1 cm after a correct response and increased by 0.3 cm after an incorrect response). This allowed for a faster decrease at the beginning of the trial when fewer errors were made and more resolution when the task began to approach the threshold for detection (< 1.0 cm). The 3:1 ratio of amplitude increase to decrease is necessary to counter the fact that half the time the subject will guess correctly even below their threshold for sensing motion. Minimum displacement size was set at 0.1 cm, however, due to physical properties of the motor and position controller the actual displacements could be slightly smaller or larger (Fig. 1d). Once a decision was made, the participant moved the non-dominant arm back to the middle segment of the rectangle to initiate the next trial. The MDT task was then repeated to examine test-retest reliability.

### Data Processing and Analysis

Position and velocity of the robot handles were recorded at a sampling rate of 1000 Hz. Signals were filtered using a sixth-order double-pass Butterworth low-pass filter with a cutoff frequency of 10 Hz. All data were collected using the Dexterit-E software program (version 3.6.1, Kinarm, Kingston, ON, Canada). MDT task data were analyzed using custom scripts in MATLAB (version 2017a, Mathworks Inc., Natick, Massachusetts).

### MDT task parameters

Discrimination Threshold – The use of a 3:1 staircase ratio for incorrect:correct trials means that the median displacement across trials is the estimated Discrimination Threshold for that individual. Discrimination Threshold was calculated as the median of all hand displacements (leftward and rightward displacements; Fig. 1d) after the first wrong answer was recorded. Median of the displacements was chosen rather than a mean because the 3:1 asymmetry in step size would disproportionately influence the mean. Since the commanded displacements could differ from the actual displacements of the hand because of physical properties of the robot and the properties of the position controller, the actual displacements were used to calculate the Discrimination Threshold. However, the difference between commanded and actual displacement was < 1% for displacements > 1 cm, and was less than < 7% for 0.1 cm displacements.

Displacement Variability – standard deviation of hand displacements (leftward and rightward displacements). This measure captures the consistency of subject performance across the task. The standard deviation was calculated from all movements after the first wrong answer was recorded.

### Statistical analysis

Each parameter was compared between Test 1 and Test 2 to quantify test-retest reliability. Intraclass Correlation Coefficient estimates and their 95% confident intervals were calculated using MATLAB based on a single-rating (k = 1), absolute-agreement, 2-way mixed-effects model [49]. Levels of reliability followed guidelines set out by Koo and Li [50]. We also calculated the within-subjects Standard Deviation (Sw, [49]) as a measure of the precision of the test. Sw was calculated using the equation:

$$ Sw=\sqrt{\sum {\left(T1-T2\right)}^2/2n} $$

where n = number of subjects [51].

The ‘repeatability coefficient’ (CR) was calculated as an index that quantifies absolute reliability. The CR is calculated in the units of the measured parameter and is the value below which absolute differences between two measurements would lie (with 95% probability). It is calculated by multiplying the Sw by 2.77 (√2 times 1.96) [51]. Bland-Altman plots were also generated to examine learning effects.

### Reduction of number of trials

The number of trials necessary to accurately determine the sensory threshold was initially determined based on simulated data. We examined the possibility of reducing the number of trials (post-hoc) by calculating the Discrimination Threshold and Displacement Variability from a subset of the collected trials. We calculated each variable using 25, 30, 40, 50, 75, 100 and 125 trials (starting from the beginning of the task). The variables were calculated in the same way as above, using the actual arm displacement data after the first wrong answer was recorded. We used Spearman correlations to compare the calculated variables for each subset of trials with the original variables calculated from all trials. We also re-calculated the test-retest reliability for each subset of trials using ICC, Sw and CR.

### Experiment 2

**Fig. 2:**
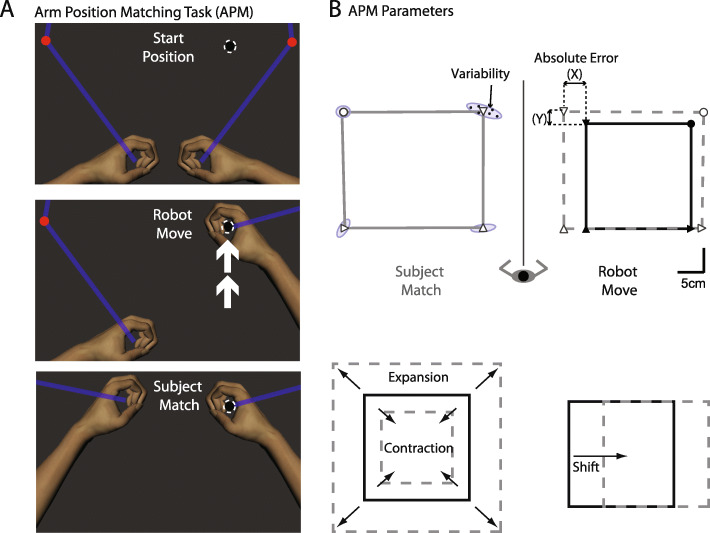
**a** Schematic of the Arm Position Matching (APM) task using the Kinarm end-point robot. The robot moved the dominant arm to one of four target locations (dashed circle) and the subject “mirror” matched with the opposite arm with vision occluded. **b** Schematic diagrams of parameters calculated for the APM task. Icons connected by the solid black lines are the 4 positions of the robot-moved hand, whereas icons connected by the solid grey lines are the positions of the subject-moved matching hand. Icons connected by grey dashed lines are the matching positions of the subject-moved hand reflected over the robot-moved hand, for comparison purposes. Different shapes (small circle and triangles) correspond to the four different target locations. Ellipses reflect the variability of the matching locations for all trials

In the APM task [10] the robot moved the dominant arm to one of 4 spatial positions in the workspace and participants were instructed to “mirror-match” their non-dominant hand to the position of their dominant hand (Fig. 2a). Once participants believed that they reached the target position, they verbally indicated to the examiner, who would then click a button on the computer screen that would signal the software to log the position of the robot handle and initiate the next trial. The task consisted of 6 blocks of trials, with 4 trials per block to yield a total of 24 trials. The APM task was then repeated to examine test-retest reliability. Note that although the MDT task results are presented first (Experiment 1), participants always completed the two repetitions of the APM task first based on a pre-determined task collection order.

### APM task parameters

The standard parameters calculated for the APM task were used in our analysis: Absolute Error, Variability, Spatial Shift and Contraction/Expansion Ratio ( [10, 52]; Dexterit-E 3.6.1, Kinarm, Kingston, ON, Canada, (Fig. 2b)). Measures were performed for the X and Y direction separately, and also combined together vectorially.

Absolute Error - calculated as a measure of accuracy. Error was calculated as the mean absolute distance error across all trials (averaged across target locations).

Variability – the standard deviations of the subject’s hand position (averaged across target locations).

Spatial Shift (Shift) – indicator of systematic errors between the arms. Shift was calculated as the mean error between the target and matched position for each target location, (averaged across target locations).

Contraction/Expansion ratio (Con/Exp) – the extent of movements made with the matching arm compared to the target movements generated by the robot.

### Statistical analysis

Each parameter was compared between Test 1 and Test 2 as in Experiment 1. The relationship between the APM and MDT parameters were compared using a Spearman’s rank-order correlation coefficient. For comparison purposes, Spearman’s rank-order correlation coefficients were also calculated between repeated tests for APM and MDT task parameters.

## Results

Twenty-one participants completed the study (18–22 years of age, 10 males, 11 females, 19 right hand dominant). Average time to complete the MDT and APM tasks were 17.9 min ± 0.79 and 1.4 ± 0.2 min, respectively, for each limb.

### Experiment 1

**Fig. 3:**
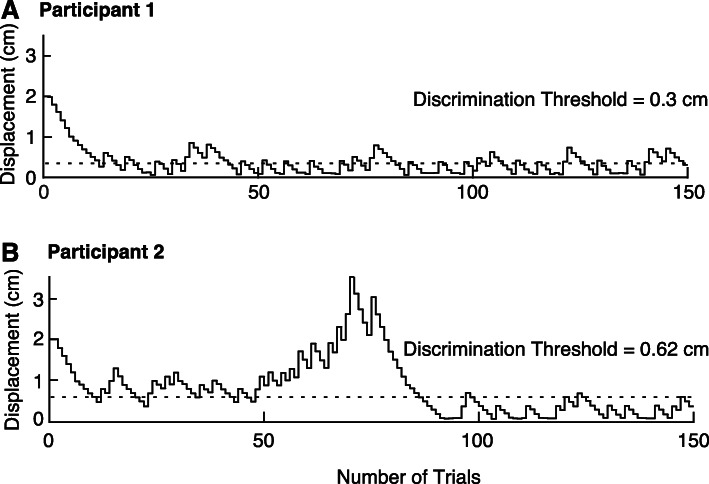
Exemplar performance of two participants on the MDT task. **a** Performance of Participant 1. Hand displacement magnitude is plotted for the entire task. Dashed line reflects the Discrimination Threshold. **b** Performance of Participant 2

The performance of two participants on the MDT task is shown in Fig. 3. Participant 1 is an individual with relatively good movement discrimination (Discrimination Threshold = 0.30 cm) and low variability (Displacement Variability = 0.20 cm). Participant 2 is an individual with relatively poor movement discrimination (Discrimination Threshold = 0.62 cm) and high variability (Displacement variability = 0.71 cm). This person displayed a large number of errors in the middle of the task leading to a large increase in the magnitude of applied displacements.

**Fig. 4:**
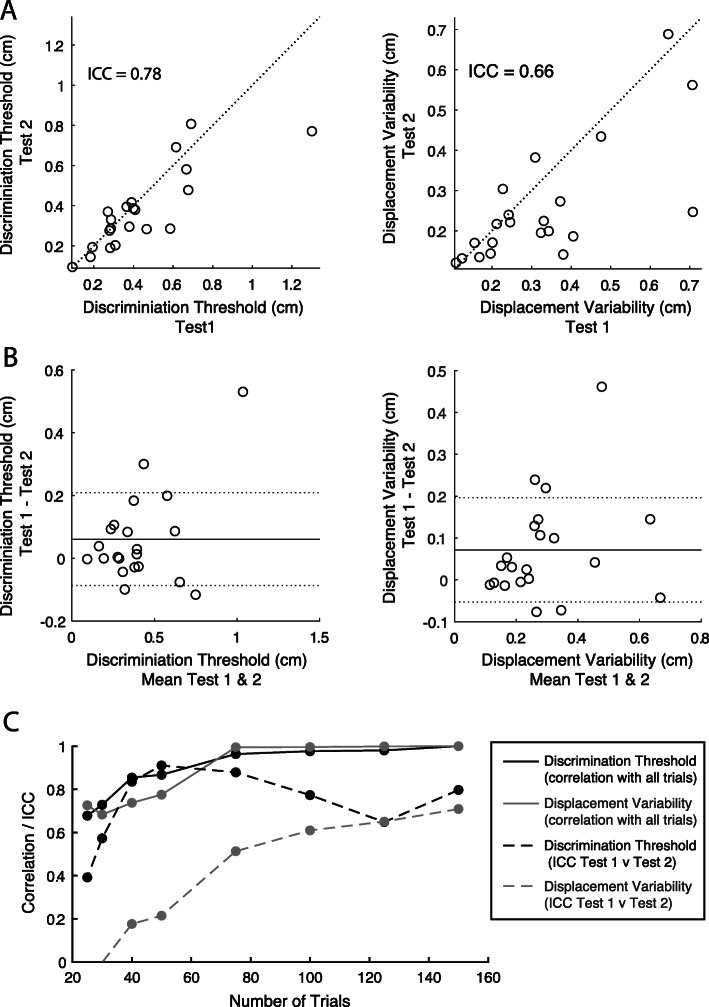
**a** Test-retest reliability comparisons for Discrimination Threshold and Displacement Variability on the MDT task. Dashed line is the unity line. **b** Bland-Altman plots of Discrimination Threshold and Displacement Variability. Mean of Test 1 and Test 2 plotted on x-axis, difference between Test 1 and Test 2 plotted on the y-axis. Solid horizontal black line reflects the mean difference and dashed lines reflect the mean+/− 1.96 standard deviations of the difference. **c** Analysis of reducing the number of trials used. Solid black line and circles denote the Spearman’s coefficient for correlations between Discrimination Threshold calculated with 150 trials and parameters calculated using 25, 40, 50, 75, 100, 125 and 150 trials. The same comparison is plotted for Displacement Variability using the grey circles/line. Dashed black line and circles represent the ICC values for Discrimination Threshold calculated for Test 1 and Test 2 using 25, 40, 50, 75, 100, 125 and 150 trials. The same comparison is plotted for Displacement Variability using the grey dashed line and circles

Group median values for Discrimination Threshold were 0.38 cm for Test 1 and 0.34 cm for Test 2 (Table 1). There was no statistical difference between Discrimination Thresholds when data for left and right directions were separated (paired t-test, *p* > 0.05) Group median for Displacement Variability was 0.31 cm for Test 1 and 0.22 cm for Test 2. Discrimination Threshold between Test 1 and Test 2 had an ICC of 0.78 (0.52–0.9) which is considered moderate to excellent (Table 1, Fig. 4a). The Displacement Variability had an ICC of 0.66 (0.28–0.90), which is considered poor to good. Bland-Altman plots are shown in Fig. 4b. Mean difference between Test 1 and Test 2 was positive for both parameters, indicating that on average the parameter values for Test 1 were higher. This difference was not statistically significant for Discrimination Threshold (mean difference Test 1-Test 2 = 0.06 cm; Wilcoxon Signed Rank test, Test 1 vs Test 2, *p* = 0.14). The difference was statistically significant for Displacement Variability (mean difference Test 1-Test 2 = 0.09 cm; Wilcoxon Signed Rank test, Test 1 vs Test 2, *p* = 0.028). For both Test 1 and Test 2, Discrimination Threshold and Displacement Variability were significantly correlated (T1, Spearman’s r = 0.75, *p* < 0.001; T2, Spearman’s r = 0.89, p < 0.001).

**Table 1:** MDT task parameter values and inter-rater reliability

Task	Parameter	Median		Test-Retest (T1 v T2)				Correlation (r) between all trials & 50 trials	
		T1 (cm)	T2 (cm)	ICC (95% CI) Absolute	Correlation (r)	Sw (cm)	CR (cm)	T1	T2
MDT Task	Discrimination Threshold	0.38	0.34	0.78 (0.52–0.90)	0.8**	0.111	0.306	–	–
	Displacement Variability	0.31	0.22*	0.66 (0.28–0.86)	0.71**	0.100	0.276	–	–
MDT Task (50 Trials)	Discrimination Threshold	0.40	0.37	0.90 (0.77–0.96)	0.90**	0.088	0.244	0.87**	0.88**
	Displacement Variability	0.26	0.19	0.20 (−0.22–0.57)	0.20	0.173	0.480	0.78**	0.89**

*Sw* Within-subjects standard deviation

*CR* Repeatability coefficient

* *p* value < 0.05

**bonferroni-corrected *p* value < 0.001

The results of the trial reduction analyses revealed that Discrimination Threshold and Displacement Variability were well-established for most participants after only 50 trials (Fig. 4c, Table 1). Group median values for Discrimination Threshold were 0.40 cm for Test 1 and 0.38 cm for Test 2 after 50 trials. Group median for Displacement Variability was 0.26 cm for Test 1 and 0.19 cm for Test 2 after 50 trials (Table 1). Discrimination Threshold and Displacement Variability were highly correlated with the same variables calculated using only 50 trials (or fewer, depending on when the first wrong answer was recorded; r = 0.78–0.89; Fig. 4c, Table 1). Interestingly, ICC values for test-retest reliability were highest for 50 trials for Discrimination Threshold and was found to be good to excellent (ICC (C,1) = 0.90 (0.77–0.96); Fig. 4c, Table 1). ICC values for Displacement Variability were much lower and generally poor with fewer trials and continued to rise with the inclusion of more trials.

### Experiment 2

**Fig. 5:**
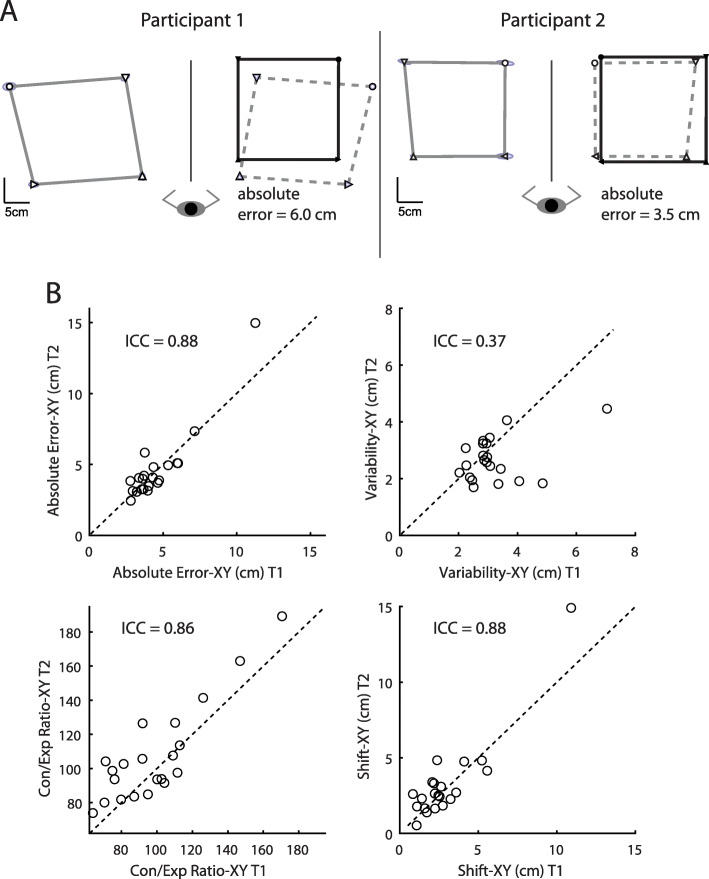
**a** Performance of exemplar participants on the APM task. Participant 1 is shown in the left traces and Participant 2 is shown in the right traces (note: participants are the same ones depicted in Fig. 3). Icons and lines same as in top half of Fig. 2b. **b** Inter-rater reliability for APM parameters between Test 1 and Test 2. Dashed line is the unity line

All parameter directions were analyzed (X, Y and combined XY) but almost identical results were found for each parameter, and thus, only XY parameters are presented here. The performance of two participants on the APM task is shown in Fig. 5 and are the same participants shown in Fig. 3. Participant 1 is an individual with relatively poor position sense (Absolute Error-XY = 6.0 cm, Variability-XY = 2.2 cm, Shift-XY = 5.6 cm, Con/Exp-XY = 1.12) but good movement discrimination (Fig. 3, Participant 1). Participant 2 is an individual with good position sense (Absolute Error-XY = 3.5 cm, Variability-XY = 2.6 cm, Shift-XY = 2.3 cm, Con/Exp-XY = 0.82), but poor movement discrimination (Fig. 3, Participant 2).

Group median parameter values for APM Test 1 and Test 2 as well as ICC values are presented in Table 2. In the APM task, Absolute Error, Con/Exp ratio and Spatial Shift were all excellent based on the mean of the ICCs, but the lowest range for the confident interval for these measures dropped down into the moderate range. The level or reliability for Variability was poor to moderate (Variability-XY ICC = 0.37).

**Table 2:** APM Task Parameter Values and Inter-rater reliability

Task	Parameter	Median		Test-Retest (T1 v T2)			
		T1	T2	ICC (95% CI) Absolute	Correlation (r)	Sw	CR
APM Task	Absolute Error–XY (cm)	3.95	3.99	0.88 (0.74–0.95)	0.88**	0.781	2.16
	Variability–XY (cm)	2.95	2.58	0.37 (−0.09–0.63)	0.33	0.812	2.249
	Con/Exp Ratio–XY	0.95	0.99	0.86 (0.65–0.94)	0.88**	0.104	0.288
	Spatial Shift–XY (cm)	2.38	2.60	0.87 (0.72–0.95)	0.88**	0.929	2.572

*Sw* Within-subjects standard deviation

*CR* Repeatability coefficient

* *p* value < 0.05

**bonferroni-corrected *p* value < 0.001

### Comparison between MDT and APM tasks

**Fig. 6:**
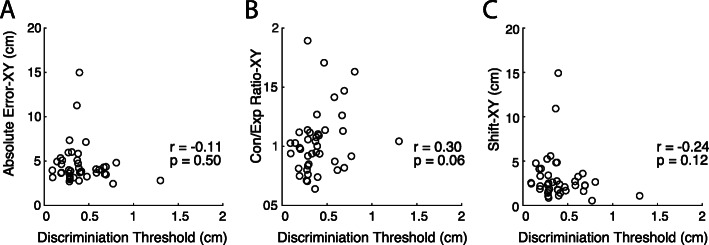
Comparisons between Median Displacement from the MDT task with all 150 trials, and (**a**) Absolute Error-XY, **b** Cont/Exp Ratio-XY, and (**c**) Shift-XY from the APM task; r value is Spearman’s rank-order correlation coefficient. No significant relationships were found (*p* > 0.05)

Spearman’s correlations were performed for all parameter pairings between the two tasks and are reported in Table 3 and shown in Fig. 6. *P*-values presented are uncorrected for multiple comparisons. All comparisons found low to negligible correlations [53] none of which were statistically significant even before correction for multiple comparisons. Comparisons between MDT Discrimination Threshold and APM parameters produced correlation coefficients ranging from − 0.24 to 0.30. We also performed Spearman’s correlations on the MDT parameters calculated for the first 50 trials to ensure no relationships were found when using fewer trials. The correlation coefficients were almost identical, low to moderate correlations, none of which were significant (Table 3).

**Table 3:** MDT & APM between-task correlations

Spearman Correlation (r-values, *p*-values)			
		MDT Task	
		Discrimination Threshold	Displacement Variability
APM Task	Absolute Error–XY (cm)	r = −0.11 (*p* = 0.50)	r = − 0.07 (*p* = 0.66)
	Variability–XY (cm)	r = − 0.12 (*p* = 0.34)	r = − 0.03 (*p* = 0.83)
	Con/Exp Ratio–XY	r = 0.30 (*p* = 0.06)	r = 0.05 (*p* = 0.76)
	Spatial Shift–XY (cm)	r = − 0.24 (*p* = 0.12)	r = − 0.26 (*p* = 0.09)
		MDT Task – 50 trials	
APM Task	Absolute Error–XY (cm)	r = − 0.094 (*p* = 0.55)	r = − 0.11 (*p* = 0.49)
	Variability–XY (cm)	r = − 0.14 (*p* = 0.37)	r = − 0.075 (*p* = 0.64)
	Con/Exp Ratio–XY	r = 0.27 (*p* = 0.08)	r = 0.006 (*p* = 0.97)
	Spatial Shift–XY (cm)	r = −0.24 (p = 0.12)	r = − 0.27 (p = 0.08)

## Discussion

In this study we develop a Movement Discrimination Threshold (MDT) task designed to test the lowest amplitude movement that participants are able to reliably discriminate. We found that in a young, healthy population, the test-retest reliability for the Discrimination Threshold was moderate to excellent. For the APM task, test-retest reliability for three of the four task parameters also were moderate to excellent (Absolute Error, Spatial Shift and Exp/Cont). However, there was essentially no relationship between performances across these two tasks.

The MDT utilized an automated staircase search method that adjusted movement magnitude based on the subject’s performance. This approach offers two advantages over previously used methods. First, by having the movement magnitude automatically adjust trial-by-trial, there are no ceiling effects in the task which are a risk for similar methods that test within a fixed range of displacements (i.e. 0-1 cm) [46]. Second, this method reduces the testing time as it is able to quickly establish the threshold “online” and not rely on many trials at each specified displacement with many trials far from the subject’s threshold. The full MDT task took ~ 17 min to complete whereas previous robotic tasks using a similar approach have reported testing times of 23–45 min [43, 46].

The Discrimination Threshold was moderate to excellent test-retest reliability, but Displacement Variability was not as good. The lower ICC values for Displacement Variability are likely due to slight improvements in performance between Test 1 and Test 2, as the Displacement Variability decreased significantly between the two test times by an average of 6 mm. This could indicate a slight learning effect between two sessions of the task.

In an attempt to reduce MDT task time, we performed an additional analysis to compare parameters calculated with fewer overall trials. The results showed that the calculated parameters were well established after only 50 trials for most individuals and were highly correlated with parameters calculated using all 150 trials. The average difference between Discrimination Thresholds calculated with 50 and 150 trials is 0.018 cm, which is actually less than the average test-retest difference calculated for Discrimination Threshold (0.06 cm). Further, the ICC values between test-retest seem to improve with fewer trials, peaking at 50 trials for Discrimination Threshold (good to excellent). This may be due to the effects of fatigue or lapses in concentration during the task. In many of the displacement traces there is evidence of more variable displacement traces after 50 trials (see Figs. 1 and 3). Although the ICCs of Displacement Variability are not as strong when using 50 trials, the primary outcome measure from the MDT task is Discrimination Threshold, and therefore, the priority should be to optimize test-retest reliability in this parameter while reducing task time as much as is reasonably possible. Reducing the number of trials to 50 would be advantageous as it would dramatically reduce task time to ~ 5 min per arm.

Our task is similar to a recently developed robotic Arm Movement Detection (AMD) task which reduced task time to under 15 min by using graded force perturbations applied to a robotic handle held by participants [45]. In the AMD task the force perturbation is increased or decreased and participants are asked to respond to the question “do you feel the perturbation” and a Proprioception Acuity Score is calculated from the threshold and variability for movement detection from 10 trials. Group differences were found between controls and participants with stroke, and also between stroke participants with and without clinically-determined proprioceptive loss, providing a promising clinical tool for assessment of proprioception following stroke. A key difference in our task is the assessment of movement discrimination (was the movement left or right?) compared to movement detection in the AMD task (did you feel a movement, yes or no?). However, since both tasks are related to detection thresholds there may be a correlation in performance across these tasks in healthy subjects. In contrast, we predict that there would be no correlation between the AMD task and our APM task given the lack of a correlation between MDT and APM tasks.

All APM parameters had good to excellent inter-rater reliability except for Variability which had a low, non-significant inter-rater relationship. The low inter-rater value for Variability was somewhat surprising given that the inter-rater reliability found previously for this parameter was excellent (r = 0.81) [10]. This discrepancy is likely due to the fact that the current analysis only included healthy young participants, whereas the previous study included neurological intact controls and individuals with stroke [10]. The between-subject variability of control participants is quite low and forms a cluster at the lower (better) end of performance. Patient data spans a larger range which improves inter-rater scores [54]. This effect was also observed for the variability parameters of a robotic kinesthesia task where ICC values for controls only ranged from 0.10–0.52 but improved to 0.80–0.94 when stroke participant data were added [55]. The key issue is that low inter-rater reliability scores based on the performance of healthy controls does not mean a task is not clinically useful, as has been suggested [56]. Between-subject variability in healthy subject performance may be too low to consistently reproduce performance [41]. However, variability in performance may be much larger in a given patient population, and thus, may lead to improved reliability in that population.

We were surprised to find no significant correlations between any parameters across the two tasks. We expected that if a subject had a lower movement discrimination threshold, they would perform better on a position matching task, and vice versa. One obvious difference between the two tasks is that the APM task involves the use of both arms, whereas the MDT task relies primarily on one arm. In the APM task the robot moved the dominant arm and the participants were required to use of the opposite arm to mirror match the position. This requires interhemispheric communication and likely transfer of proprioceptive input between hemispheres [57, 58]. Indeed, position matching tasks that involve the same arm (ipsilateral matching) show different brain activity [56] and a reduction in absolute error [39] compared to tasks that involve the use of both arms. Therefore, it may be that performance on the current tasks would have been more closely related if they both involved the use of a single limb. However, a previous study that did compare single-arm proprioceptive acuity (the ability to discriminate between two different positions) with performance on a single-arm position matching task, also found that the two were not related [42]. This suggests that while some differences in task performance may stem from the involvement of one arm compared to two, this likely does not account for all of the differences in performance.

It is possible that the tasks in the current study assess two different aspects of proprioception that are not necessarily related, or at least not always in the way that one might intuitively expect. The arm position matching task measures the ability to perceive limb position. In contrast, the movement detection task measures the ability to perceive a change in position which his more related to kinesthesia. An interesting early study of the relationship between proprioceptive acuity and joint position sense compared healthy individuals to trained ballet dancers and found that ballet training had what the authors called a “paradoxical” effect on proprioception [59]. While the onset to detection of passive movement was quicker in the dancers, signaling enhanced proprioceptive acuity, they also displayed larger errors in the position match task compared to the healthy individuals. This highlights that individual differences such as training history, may lead to unique and somewhat unexpected relationships between proprioceptive acuity and joint position matching.

### Clinical implications

The fact that performance on each type of task was not correlated highlights that several different measures may be required to obtain a full picture of proprioceptive performance within an individual. Previous work has highlighted that several participants with stroke who were impaired on a between-limb position matching task, were not impaired on a within-limb position matching task [60]. Having a “suite” of proprioceptive tasks will help to tease out which aspects of proprioception are impaired in patient populations. However, it is also likely that higher correlations between the two current tasks will be found for certain patients or patient populations, as deficits in these two aspects of proprioception due to neurological injury or disease could impact common neural circuits that support different aspects of proprioceptive function [9]. Future work will examine the relationship between these two tasks with advancing age and with neurological deficits as performance in the APM task is influenced by age [52] and by diseases such as stroke [10].

One limitation of the current paradigm was that the tasks were not presented in a randomized order, participants always completed the APM test-retest prior to the MDT test-retest. However, it is unlikely that this influenced performance on either task. Participants were given adequate rest between all tasks, so fatigue should not affect task performance. Motivation or inattention may be a concern given that the task is not that engaging and requires a lot of focus to sense small displacements of the limb for many trials. For example, Participant 2 in Fig. 3 displayed an increase in the number of errors from trial 50 to 75 highlighting the benefit of reducing the task to 50 trials. Another limitation when comparing the two tasks may be the use of median to determine the Discrimination Threshold (MDT) vs the mean to determine Absolute Error (APM). While there is no reason that the use of median for one parameter and mean for the other should substantively impact the results, in the future it may be beneficial to have the same metric for both tasks for comparative purposes. Finally, the current work is also limited to a young, healthy control sample to rule out variability of the results due to age [52, 61] and the number of subjects is less than ideal [50].

## Conclusion

We found that the MDT task is a feasible and reliable assessment tool to determine the threshold for movement discrimination. The use of a staircase search method for determining the threshold based on subject performance shortens the task to under 20 min, with the potential to reduce to 5–6 min per arm with the use of 50 trials instead of 150 trials. Performance on the MDT task was not related to a mirror matching proprioceptive task (APM), suggesting that proprioceptive acuity (via movement discrimination) is not necessarily related to accuracy on a joint position matching task, and that these abilities may be separate components of proprioception. These tasks were designed for the assessment of proprioception following neurological disease or injury, and the current findings highlight the potential importance of using different tasks in order to capture different facets of proprioceptive function.

## Data Availability

The data that support the findings of this study are available from the corresponding author upon request.

## Abbreviations

MDT: Movement Discrimination Threshold
APM: Arm Position Matching
ICC: Intra-class Correlation Coefficient
AMD: Arm Movement Detection
